# CTRP12 ameliorates atherosclerosis by promoting cholesterol efflux and inhibiting inflammatory response via the miR-155-5p/LXRα pathway

**DOI:** 10.1038/s41419-021-03544-8

**Published:** 2021-03-10

**Authors:** Gang Wang, Jiao-Jiao Chen, Wen-Yi Deng, Kun Ren, Shan-Hui Yin, Xiao-Hua Yu

**Affiliations:** 1 Department of Cardiology, The First Affiliated Hospital of University of South China, Hengyang, 421001 Hunan China; 2 Institute of Clinical Medicine, The Second Affiliated Hospital of Hainan Medical University, Haikou, 570100 Hainan China; 3 Department of Pathophysiology, School of Basic Medical Sciences, Anhui Medical University, Hefei, 230032 Anhui China; 4 Department of Neonatology, The First Affiliated Hospital of University of South China, Hengyang, 421001 Hunan China

**Keywords:** Non-coding RNAs, Cardiovascular diseases

## Abstract

C1q tumor necrosis factor-related protein 12 (CTRP12), a conserved paralog of adiponectin, is closely associated with cardiovascular disease. However, little is known about its role in atherogenesis. The aim of this study was to examine the influence of CTRP12 on atherosclerosis and explore the underlying mechanisms. Our results showed that lentivirus-mediated CTRP12 overexpression inhibited lipid accumulation and inflammatory response in lipid-laden macrophages. Mechanistically, CTRP12 decreased miR-155-5p levels and then increased its target gene liver X receptor α (LXRα) expression, which increased ATP binding cassette transporter A1 (ABCA1)- and ABCG1-dependent cholesterol efflux and promoted macrophage polarization to the M2 phenotype. Injection of lentiviral vector expressing CTRP12 decreased atherosclerotic lesion area, elevated plasma high-density lipoprotein cholesterol levels, promoted reverse cholesterol transport (RCT), and alleviated inflammatory response in apolipoprotein E-deficient (apoE^−/−^) mice fed a Western diet. Similar to the findings of in vitro experiments, CTRP12 overexpression diminished miR-155-5p levels but increased LXRα, ABCA1, and ABCG1 expression in the aortas of apoE^−/−^ mice. Taken together, these results suggest that CTRP12 protects against atherosclerosis by enhancing RCT efficiency and mitigating vascular inflammation via the miR-155-5p/LXRα pathway. Stimulating CTRP12 production could be a novel approach for reducing atherosclerosis.

## Introduction

Atherosclerosis, the pathological basis of most cardiovascular disease, is a chronic vascular inflammatory disease characterized by dysregulated lipid homeostasis^1^. As a major cell type in the atherosclerotic lesions, macrophages play a central role in the occurrence and development of atherosclerosis. During atherogenesis, circulating monocytes transmigrate into the subintima where they differentiate into macrophages. After uptake of large amounts of modified lipoproteins, such as oxidized low-density lipoprotein (ox-LDL), macrophages are transformed into lipid-rich foam cells, a hallmark of early-stage atherosclerotic lesions^2^. As a transmembrane protein, the primary function of ATP binding cassette (ABC) transporter A1 (ABCA1) is to mediate cholesterol efflux to lipid-poor apolipoprotein A-I (apoA-I) for generation of nascent high-density lipoprotein (HDL) particles. ABCG1, an important member of the ABCG subfamily, promotes the efflux of intracellular cholesterol to HDL. Decreased cholesterol efflux mediated by ABCA1 and ABCG1 is regarded as a critical mechanism for macrophage foam cell formation^3–5^. Studies from our groups and others have revealed that increased ABCA1 and ABCG1 expression contributes to alleviation of lipid accumulation and atherosclerotic lesions in atherosclerosis-prone mice^6,7^. Moreover, macrophage foam cells can secrete a variety of pro-inflammatory mediators, leading to further development of atherosclerosis^8^. Thus, a better understanding of the regulatory mechanisms for cholesterol efflux and vascular inflammation is important to develop novel therapeutic strategies for atherosclerosis.

ABCA1 and ABCG1 are regulated by a variety of bioactive molecules. Liver X receptor α (LXRα, encoded by the *NR1H3* gene), a nuclear hormone receptor, is thought to be the most important transcription factor to induce ABCA1 and ABCG1 expression^9^. We and others previously reported that administration of leonurine, kuwanon G or dihydromyricetin inhibits lipid accumulation in THP-1 macrophages and protects against atherosclerosis in mice by activating the LXRα-ABCA1/ABCG1 signaling pathways^10–12^. In addition to its role in the regulation of lipid metabolism, activation of LXRα can suppress inflammatory signaling in macrophages^13^. MicroRNAs (miRNAs) are a class of small noncoding RNA molecules that can post-transcriptionally modulate the expression of their downstream target genes. There is accumulating evidence that dysregulation of miRNAs is associated with lipid metabolism disorder, inflammatory response, and atherosclerosis progression^14,15^. MiR-155-5p is located in a region called B cell integration cluster on human chromosome 21^16^. Plasma miR-155-5p levels are significantly elevated in rheumatoid arthritis patients with subclinical atherosclerosis as compared to healthy volunteers^17^. Increased levels of circulating miR-155-5p are also observed in patients with unstable coronary artery disease^18^. Importantly, there is a negative correlation between circulating adipocyte-derived extracellular vesicle miR-155-5p isolated from adolescent obesity subjects and cholesterol efflux capacity in THP-1 macrophages^19^. Knockdown of miR-155-5p inhibits pro-inflammatory cytokine production and ameliorates lipopolysaccharide (LPS)-induced acute lung injury in mice^20^. These findings suggest that miR-155-5p plays a role in the regulation of lipid metabolism and inflammation. It is still unclear, however, whether miR-155-5p affects these two processes by reducing the activity of LXRα.

C1q tumor necrosis factor-related protein 12 (CTRP12), also known as adipolin, is a newly discovered adipokine and a conserved paralog of adiponectin^21^. Overexpression of CTRP12 was shown to ameliorate cardiomyocyte injury induced by LPS^22^. Deletion of CTRP12 in mice aggravates neointimal thickening after vascular injury^23^. In addition, circulating CTRP12 levels are significantly decreased in patients with coronary artery disease and show an independent correlation with the risk of this disease^24^. These findings suggest that CTRP12 is associated with cardiovascular disease. However, its role in atherogenesis is still unclear. In the present study, we demonstrated for the first time that CTRP12 mitigates atherosclerosis by promoting ABCA1/ABCG1-dependent cholesterol efflux and inhibiting inflammatory response via the miR-155-5p/LXRα signaling pathway, thereby providing a novel target for the prevention and treatment of atherosclerotic cardiovascular disease.

## Materials and methods

### Mice, diet and treatment

Forty-five male 8-week-old apoE^−/−^ ﻿mice on a C57BL/6 background were purchased from Changzhou Cavens Lab Animal ﻿Co., Ltd (Jiangsu, China), ﻿and housed under a 12 h light/dark cycle in sterilized filter-topped cages with free access to food and water with a constant humidity (55 ± 5%) and temperature (23 ± 1 °C). These mice were randomly divided into control group, LV-NC group, and LV-CTRP12 group with 15 animals in each group. Mice were fed a Western diet (21% fat, 0.3% cholesterol; Research Diets) for 12 weeks and simultaneously injected with PBS, LV-NC, or LV-CTRP12 (2 × 10^9^ TU/mL) via the tail vein once every 3 weeks. At the end of the study, mice were sacrificed and the heart, aorta, and blood samples were collected. All surgeries were conducted under sodium pentobarbital anesthesia. The animal experiments were approved by the Institutional Animal Care and Use Committee of the Second Affiliated Hospital of Hainan Medical University.

### THP-1 cell culture, mouse peritoneal macrophage (MPM) isolation, and CTRP12 overexpression

THP-1 monocytes (TIB-202, American Type Culture Collection) were cultured in RPMI 1640 medium (Sigma-Aldrich, St. Louis, MO, USA) supplemented with 10% fetal bovine serum (FBS, Sigma-Aldrich) and 1% penicillin-streptomycin ﻿(Beyotime, Shanghai, China) at 37 °C in a humidified atmosphere of 5% CO_2_. To induce the differentiation of monocytes into macrophages, cells were treated with 100 nM phorbol 12-myristate 13-acetate (PMA, Sigma-Aldrich) for 24 h. Then, macrophages were incubated with 50 µg/mL ox-LDL (Yiyuan biotechnology, Guangzhou, China) for 48 h to become foam cells. Lentiviral vector expressing CTRP12 (LV-CTRP12) and empty vector (LV-NC) were provided by Genechem (Shanghai, China). THP-1 macrophage-derived foam cells were transfected with LV-CTRP12 or LV-NC at a multiplicity of infection of 100 in the presence of 8 mg/mL of polybrene for 24 h. Cells in control group were incubated with PBS only. Thereafter, cells were cultured in fresh RPMI 1640 medium containing 10% FBS for 48 h. Western blot was performed to assess transfection efficiency.

MPMs were extracted as previously described^25^. Briefly, mice (*n* = 5 in each group) were intraperitoneally injected with 3% thioglycolate three days prior to sacrifice. MPMs were isolated from the mouse peritoneal cavity with PBS. After centrifugation (300 rpm, 5 min, 4 °C), cells were cultured in RPMI 1640 medium containing 10% FBS and 1% penicillin-streptomycin.

### Evaluation of intracellular lipid droplets by Oil red O staining

After treatment with PBS, LV-NC or LV-CTRP12, THP-1 macrophage-derived foam cells were fixed in 4% paraformaldehyde for 5 min and rinsed several times in PBS. These cells were then stained with Oil Red O working solution for 5 min at 37 °C in the dark, and destained with 60% isopropanol for 10 s. The stained cells were photographed under an inverted microscope (Olympus B×50).

### Detection of intracellular cholesterol and triglyceride (TG) contents

High-performance liquid chromatography was used to measure intracellular cholesterol amounts as previously described^26^. Briefly, cells were washed in PBS and lysed by sonication on ice using an ultrasonic processor (Scientz, Zhejiang, China). Protein concentrations in cell lysate supernatants were determined using a BCA protein assay kit (Beyotime). The cell lysates were supplemented with isovolumetric 15% KOH (diluted with 150 g/L ethanol) and vortexed. Cellular lipids were then extracted with n-hexane-isopropanol (3:2, V/V) and dissolved in isopropanol (50 mg/mL). Cholesterol standard calibration solution ranging from 0 to 50 mg/mL was prepared. The reaction mixture (Tris-HCl (500 mM, pH = 7.4), MgCl_2_ (500 mM), dithiothreitol (10 mM) and 5% NaCl), was added into 0.1 mL of cell solution or cholesterol standard calibration solution. The amount of total cholesterol (TC) was detected by adding 0.4 U cholesterol oxidase combined with 0.4 U cholesterol esterase, and free cholesterol (FC) content was determined by supplementing 0.4 U cholesterol oxidase alone. Each reaction tube was incubated at 37 °C for 30 min, and the reaction was terminated by adding 100 μL of ethanol: methanol (1:1 V/V). After centrifugation, the supernatants were collected and analyzed using 2790 Chromatographer (Waters, MA, USA). Absorbance at 216 nm was monitored. Data were analyzed by the TotalChrom software (PerkinElmer, MA, USA). In addition, the TG concentration was determined using a commercial kit (Sigma-Aldrich) according to the manufacturer’s protocol.

### Cholesterol efflux assay

THP-1 macrophage-derived foam cells were plated in six-well plates and loaded with 1 μg/mL NBD-cholesterol (Invitrogen, Carlsbad, CA, USA) for 4 h. Cells were washed, equilibrated, and maintained with serum-free RPMI 1640 medium containing 0.1% bovine serum albumin (BSA) for 2 h. Then, cells were incubated with 25 μg/mL apoA-I (Sigma-Aldrich) or 50 μg/mL HDL (Sigma-Aldrich) at 37 °C for 4 h. The medium was collected, and the cells were lysed using 0.3 M NaOH solution. The fluorescence density in the medium and cell lysates was determined using a microplate spectrophotometer. Cholesterol efflux was expressed as percent fluorescence density in medium relative to total fluorescence density (medium + cells).

THP-1 macrophages were incubated with 50 µg/mL acetelyated LDL (ac-LDL, Yiyuan biotechnology) and 5 µCi/mL [^3^H]-cholesterol (PerkinElmer) for 24 h. Cells were washed with PBS and then cultured in RPMI 1640 medium containing 10 µM avasimibe (Med Chem Express, NJ, USA) and 0.5 mM 8-bromo-cAMP (8-Br-cAMP, Sigma-Aldrich). After 24 h, cells were washed with PBS and maintained in 2 mL of RPMI 1640 medium containing 1 mg/mL BSA, 25 μg/mL apoA-I, or 50 μg/mL HDL for up to 9 h. The Radioactivity of [^3^H]-cholesterol in the medium and cells was measured using a liquid scintillation counter. The percent cholesterol efflux was calculated as the ratio of radioactivity in the medium to total radioactivity (medium + cells).

### Dil-ox-LDL uptake

THP-1 macrophage-derived foam cells were treated with PBS, LV-NC, or LV-CTRP12, followed by incubation with 10 µg/mL Dil-ox-LDL (Yiyuan biotechnology) at 37 °C for 4 h. After washing with PBS three times, the cells were viewed and imaged by a fluorescence microscope.

### Transfection of LXRα small interfering RNA (siRNA) and miR-155-5p mimic/inhibitor

LXRα siRNA (forward, 5′-GGAUGCUAAUGAAACUGGUTT-3′; reverse, 5′-ACCAGUUUC AUUAGCAUCCGT-3′) and scrambled siRNA as a negative control (forward, 5′-UUCUCCGAA CGUGUCACGUTT-3′; reverse, 5′-ACGUGACACGUUCGGAGAATT-3′) were designed and synthesized by GenePharma (Shanghai, China). The siRNAs (100 nM) were transfected into lipid-enriched macrophages using Lipofectamine^®^ 3000 (Invitrogen) according to the manufacturer’s instructions. To determine the impact of gain and loss of function of miR-155-5p, THP-1 macrophage-derived foam cells were transfected with 50 nM of miR-155-5p mimic, miR-155-5p inhibitor or their respective negative controls (Ribobio, Guangzhou, China) using Lipofectamine^®^ 3000. At 48 h post-transfection, cells were processed for further analyses.

### Bioinformatics prediction and luciferase reporter assay

The potential targeting sequences between miR-155-5p and LXRα 3′ untranslated region (UTR) were predicted by miRDB (http://mirdb.org/miRDB/) and TargetScan (http://www.targetscan.org/). The RNAhybrid database (http://bibiserv.techfak.uni-bielefeld.de/rnahybrid/submission.htmL) was utilized to calculate the free energy score. Dual luciferase reporter assay was performed using 293T cells. Cells were seeded in a 96-well plate at a density of 15,000 cells per well and maintained in serum-free media for 2 h before transfection. Then, cells were co-transfected with miR-155-5p mimic or mimic control and reporter plasmid pmirGLO (Promega) containing predicted binding sequences or mutant sequences of LXRα with miR-155-5p using Lipofectamine^®^ 3000. After 48 h of transfection, cells were harvested, and the luciferase activity was detected using a Luciferase detection assay kit (KeyGen Biotech, Nanjing, China).

### Assessment of atherosclerotic lesions in the aortic root

Mice were euthanized, and the upper part of the heart and proximal aorta were dissected carefully. After rinsing with PBS, samples were embedded in Optimal Cutting Temperature compound (OCT, Sakura Finetek Japan Co., Ltd, Tokyo, Japan) and stored at −80 °C. Serial 8-μm-thick cryosections throughout the three aortic valves were obtained and placed on glass slides using a cryostat microtome (Leica CM3050 S). Sections were then stained with Oil Red O, hematoxylin-eosin (HE), and Masson. Quantitative analyses were performed using Image-Pro Plus 7.0 software.

### Detection of plasma lipid levels

Blood samples were collected from the retro-orbital plexus of apoE^−/−^ mice. Plasma levels of TC, HDL-cholesterol (HDL-C), low-density lipoprotein cholesterol (LDL-C), and TG were determined using the commercial kits (Sigma-Aldrich).

### In vivo reverse cholesterol transport (RCT) assay

The in vivo RCT assay was performed as previously described^27^. J774 macrophages were treated with 50 μg/mL ac-LDL and 5 μCi/mL [^3^H]-cholesterol for 48 h. The labeled cells were resuspended in ice-cold Dulbecco’s modified Eagle’s medium (DMEM, ThermoFisher, Scotts Valley, CA, USA) and intraperitoneally injected into apoE^−/−^ mice (5 × 10^6^ cells/mouse, *n* = 5 per group). At 6, 24, and 48 h after injection, plasma samples were collected and the radioactivity in 10 μL aliquots was evaluated by a liquid scintillation counter. The total feces were collected continuously until 48 h, vacuum dried and homogenized in 50% NaOH overnight. Then, 20 µL aliquots were used for scintillation counting. At the end of the study, mice were sacrificed, and the liver samples were harvested. The hepatic tissue (100 mg) was mixed with hexane/isopropanol (3:2) for 48 h and then dried overnight. Lipids were resolubilized in liquid scintillation fluid and radioactivity was counted. The RCT efficiency was estimated as the ratio of radioactivity in the plasma, liver, or feces to total radioactivity injected at baseline.

### RNA isolation and qRT-PCR

Total RNA was obtained from cultured cells and the tissues using TRIzol reagent according to the manufacturer’s instructions (ThermoFisher). The purity of the extracted RNA was assessed using a Nanodrop 3000 (ThermoFisher). The first strand of complementary DNA was synthesized using a high-capacity cDNA reverse transcription kit (Takara, Kyoto, Japan). Then, qRT-PCR was conducted utilizing SYBR^®^ Premix Ex TaqTM II reagent kit (Takara) on an ABI 7900HT Fast Real-Time PCR System (Applied Biosystems, Foster City, CA, USA) for 40 cycles (95 °C for 3 min, 90 °C for 15 s, and 60 °C for 1 min). The primer sequences for qRT-PCR are listed in Supplementary Table 1, which were designed and synthesized by Sangon (Shanghai, China). U6 was selected as internal control for miR-155-5p and β-actin for all others. The specificity of all PCR products was checked using melting curve analysis. Relative gene expression was determined by the 2^−ΔΔCt^ method.

### Western blot analysis

The cultured cells and tissues were lysed by RIPA buffer (Beyotime) mixed with 0.1 mmol/L phenylmethylsulfonyl fluoride. The concentration of total proteins in the extracts was quantified using a BCA protein assay kit. Then, proteins were subjected to SDS-PAGE, followed by immunoblotting with rabbit polyclonal antibody against CTRP12 (PA5-46452, 1:500, ThermoFisher), rabbit polyclonal antibody against ABCA1 (PA1-16789, 1:800, ThermoFisher), rabbit polyclonal antibody against ABCG1 (GTX30598, 1:500, GeneTex, Irvine, CA, USA), rabbit polyclonal antibody against CD36 (PA1-16813, 1:1000, ThermoFisher), mouse monoclonal antibody against SR-A (sc-166184, 1:500, Santa Cruz, TX, USA), rabbit polyclonal antibody against LXRα (L5044, 1:1000, Sigma-Aldrich) and rabbit monoclonal antibody against β-actin (ab115777, 1:1000, Abcam, Cambridge, MA, USA). After a series of rinses with PBS-T, the membranes were further incubated with HRP-labeled secondary antibodies (1:5000, Beyotime). The protein bands were visualized using Tanon 5500 (Shanghai, China) and BeyoECL Plus (Beyotime). The densitometry values were determined using Image J software and normalized to β-actin values.

### Enzyme-linked immunosorbent assay (ELISA)

Serum monocyte chemotactic protein-1 (MCP-1), tumor necrosis factor-α (TNF-α), and interleukin-10 (IL-10) levels were measured by the commercial ELISA kits (R&D systems, Minneapolis, MN, USA) according to the manufacturer’s protocol. In brief, 100 μL serum sample or standard preparation were added into the wells and incubated at 37 °C for 120 min. Thereafter, 100 μL biotin-conjugated antibody was added into each well. After 90 min of incubation, the substrate solution was added into each well and sustained for 30 min. After washing five times with TBS, TMB was added and sustained for 30 min in the dark. The absorbance at 450 nm was detected using the iMark™ Microplate Reader (Bio-Rad, Hercules, CA, USA).

### Statistical analysis

All data are represented as the mean ± standard deviation (SD) from three independent experiments. Differences between two groups were compared by an unpaired Student’s *t* test. One-way ANOVA was used to compare the differences among multiple groups. Statistical analyses were conducted using GraphPad Prism 9.0 software. A *P* value less than 0.05 was considered statistically significant.

## Results

### CTRP12 inhibits lipid accumulation and promotes cholesterol efflux from macrophages

**Fig. 1:**
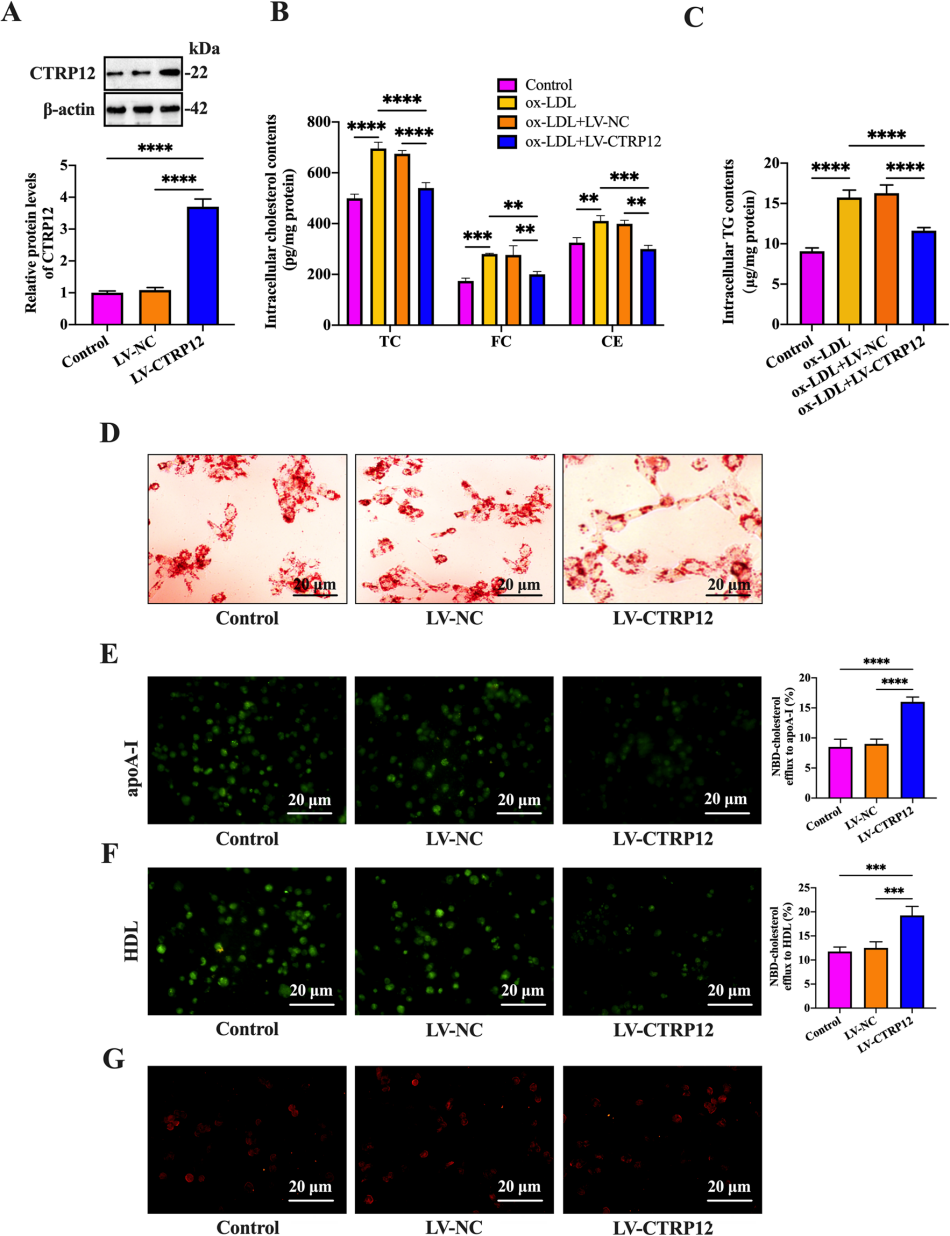
Effects of CTRP12 on cholesterol efflux and lipid accumulation in macrophages. **A**–**G** THP-1 macrophages were pretreated with or without 50 µg/mL ox-LDL for 48 h, and then transduced with PBS, LV-NC, or LV-CTRP12 for 72 h. **A** CTRP12 expression was determined by western blot. **B**, **C** Measurement of intracellular TC, FC, CE, and TG concentrations. **D** Representative images of Oil red O staining (×200). Scale bar = 20 μm. **E**, **F** Representative fluorescent images of NBD-cholesterol burden (×200). Cholesterol efflux mediated by apoA-I and HDL was quantified in these groups. Scale bar = 20 μm. **G** Representative fluorescent images of Dil-ox-LDL uptake (×200). Scale bar = 20 μm. Data are expressed as the mean ± SD from three independent experiments. **P < 0.01, ***P < 0.001, ****P < 0.0001.

Macrophage lipid accumulation is a critical event during atherogenesis. To explore the effects of CTRP12 on lipid accumulation, THP-1 macrophage-derived foam cells were treated with PBS, LV-NC, or LV-CTRP12. As shown in Fig. 1A, transfection with LV-CTRP12 elevated the protein levels of CTRP12 by ~3.5 folds compared with control group, while LV-NC did not alter its expression. In comparison with unloaded cells, THP-1 macrophage-derived foam cells displayed a significant increase in intracellular TC, FC, CE, and TG contents, and this increase was attenuated by LV-CTRP12 treatment (Fig. 1B, C). Accordingly, the Oil Red O staining results showed that CTRP12 overexpression reduced intracellular lipid droplets (Fig. 1D). It is well known that lipid accumulation is caused by decreased cholesterol efflux and/or increased cholesterol uptake^28^. Next, we investigated whether CTRP12 could affect these two processes. As expected, CTRP12 overexpression facilitated the export of NBD-cholesterol from THP-1 macrophage-derived foam cells to apoA-I (Fig. 1E) and HDL (Fig. 1F). Both avasimibe, a potent inhibitor of acyl-coenzyme A:cholesterol acyltransferase (ACAT), and 8-Br-cAMP, a cAMP activator, have been shown to enhance cholesterol efflux capacity^29,30^. In THP-1 macrophages loaded with ac-LDL, LV-CTRP12 treatment also increased the efficiency of [^3^H]-cholesterol efflux to apoA-I and HDL in the presence of avasimibe and 8-Br-cAMP (Supplementary Fig. 1). However, there was no significant difference in the uptake of Dil-ox-LDL by THP-1 macrophage-derived foam cells between control group and LV-CTRP12 group (Fig. 1G). Collectively, these results suggest that CTRP12 ameliorates lipid accumulation by promoting cholesterol efflux from macrophages.

### CTRP12 upregulates ABCA1 and ABCG1 expression in macrophages

**Fig. 2:**
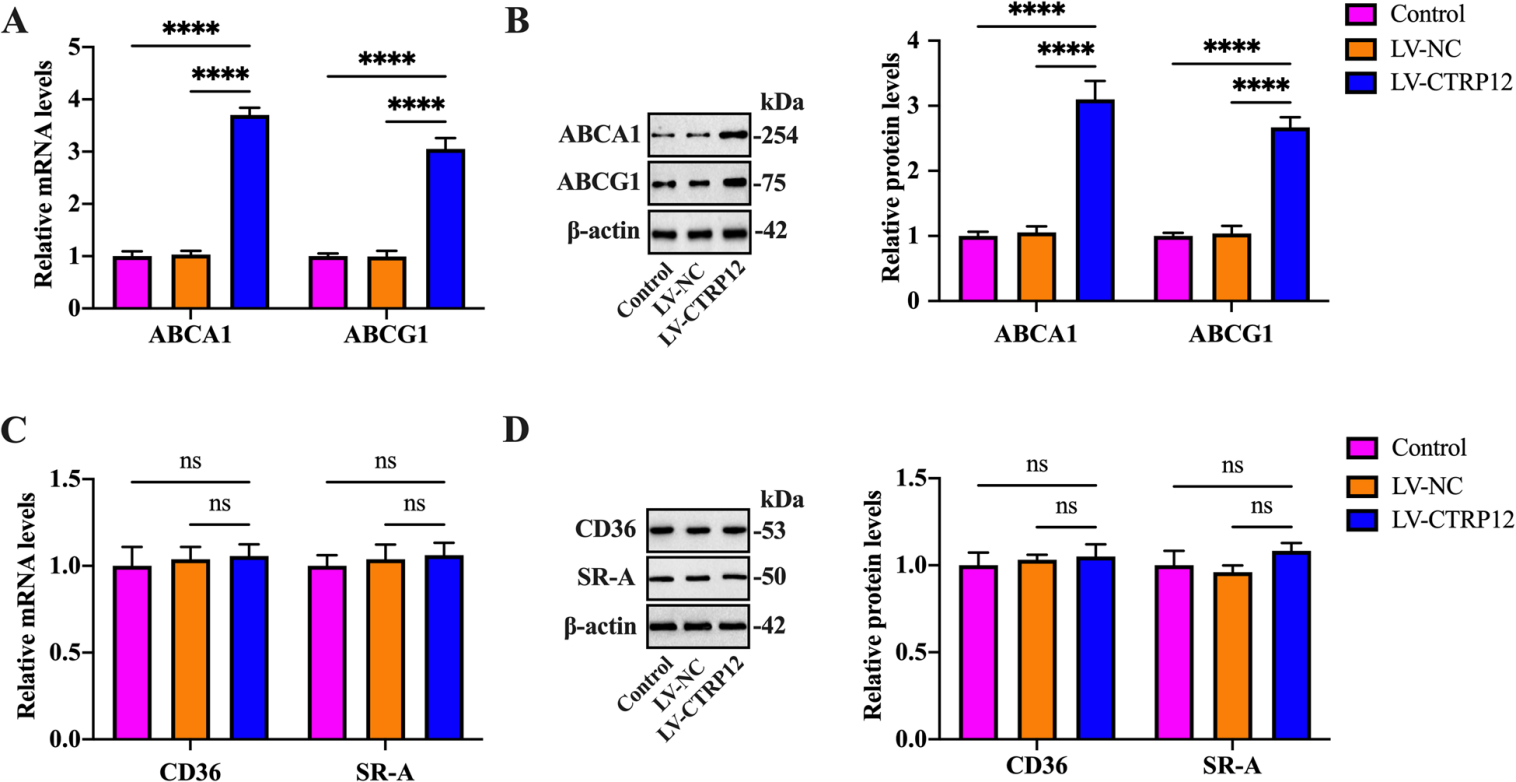
Effects of CTRP12 on the expression of cholesterol efflux and uptake markers. **A**–**D** THP-1 macrophages were incubated with 50 µg/mL ox-LDL for 48 h, followed by treatment with PBS, LV-NC, or LV-CTRP12 for 72 h. **A**, **B** The expression of ABCA1 and ABCG1 was determined by qRT-PCR and western blot. **C**, **D** Detection of CD36 and SR-A expression using qRT-PCR and western blot. Data are expressed as the mean ± SD from three independent experiments. ****P < 0.0001; ns not significant.

Intracellular cholesterol efflux is predominantly mediated by ABCA1 and ABCG1. Both CD36 and SR-A are associated with cholesterol uptake^31^. To clarify the underlying mechanisms by which CTRP12 regulates lipid accumulation, both qRT-PCR and western blot were used to measure the expression of these cholesterol efflux and uptake markers. As expected, incubation with LV-CTRP12 markedly elevated the mRNA and protein levels of ABCA1 and ABCG1 in THP-1 macrophage-derived foam cells (Fig. 2A, B). However, CTRP12 overexpression had no impact on CD36 and SR-A expression (Fig. 2C, D). These findings suggest that CTRP12 promotes cholesterol efflux by upregulating ABCA1 and ABCG1 expression.

### LXRα is involved in CTRP12-induced upregulation of ABCA1 and ABCG1 expression

**Fig. 3:**
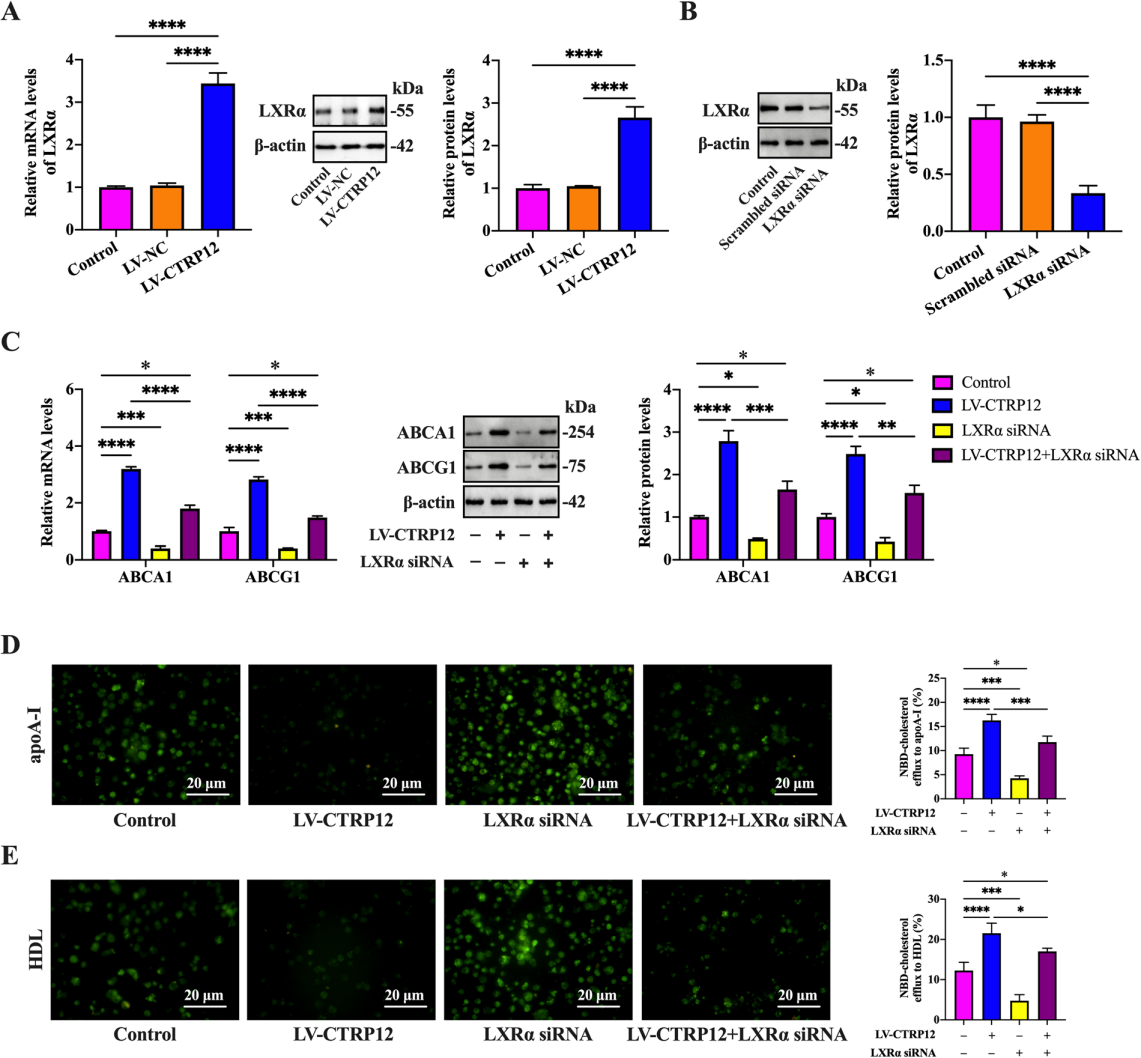
Involvement of LXRα in CTRP12-induced upregulation of ABCA1 and ABCG1 expression. **A** THP-1 macrophages were treated with 50 µg/mL ox-LDL for 48 h, followed by transfection with PBS, LV-NC, or LV-CTRP12 for 72 h. LXRα expression was determined by qRT-PCR and western blot. **B** After transfection with scrambled siRNA or LXRα siRNA for 48 h, cell lysates were immunoblotted with indicated antibodies. **C**–**E** THP-1 macrophages were loaded with 50 µg/mL ox-LDL for 48 h, transfected with LXRα siRNA for another 48 h and then treated with LV-CTRP12 for 72 h. **C** The mRNA and protein levels of ABCA1 and ABCG1 were detected by qRT-PCR and western blot, respectively. **D**, **E** Representative fluorescent images of NBD-cholesterol burden (×200) and quantitative analyses of cholesterol efflux to apoA-I and HDL. Scale bar = 20 μm. Data are expressed as the mean ± SD from three independent experiments. *P < 0.05, **P < 0.01, ***P < 0.001, ****P < 0.0001.

LXRα is the most important transcription factor to stimulate ﻿ABCA1 and ABCG1 expression. We wondered whether LXRα plays a role in the regulation of ﻿ABCA1 and ABCG1 expression by CTRP12. To this end, we first treated THP-1 macrophage-derived foam cells with LV-CTRP12 and found that CTRP12 overexpression increased the mRNA and protein levels of LXRα (Fig. 3A). Subsequently, THP-1 macrophage-derived foam cells were transfected with scrambled siRNA or LXRα siRNA. When compared with the control group, transfection with LXRα siRNA led to a marked decrease in LXRα protein expression (Fig. 3B). Finally, THP-1 macrophage-derived foam cells were transfected with LXRα siRNA, which was followed by incubation with or without LV-CTRP12. Knockdown of LXRα with siRNA reduced the effects of CTRP12 on ABCA1 and ABCG1 expression (Fig. 3C). Consistently, CTRP12-induced enhancement of NBD-cholesterol efflux to apoA-I and HDL was reversed by LXRα siRNA (Fig. 3D, E). All these findings support the notion that CTRP12 increases ABCA1 and ABCG1 expression in an LXRα-dependent manner.

### Identification of LXRα as a direct target of miR-155-5p

**Fig. 4:**
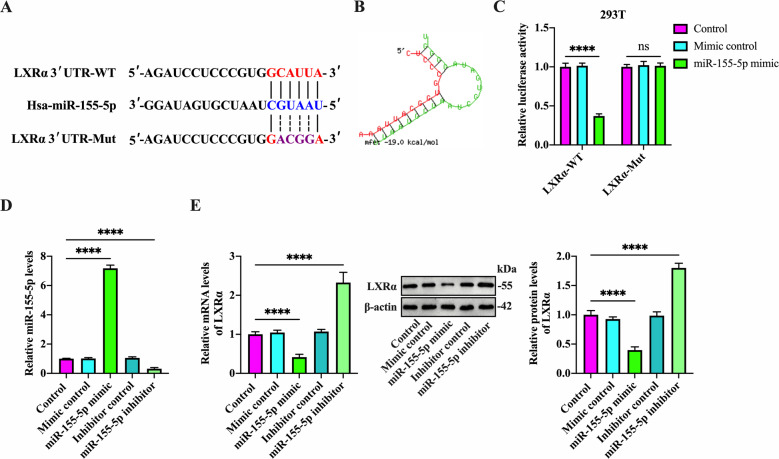
Validation of LXRα as a direct target of miR-155-5p. **A** Schematic of miR-155-5p binding site in the 3′ UTR of LXRα mRNA and corresponding mutation. **B** Calculation of free energy score by the RNAhybrid database. **C**﻿ The 293T cells were co-transfected with the luciferase reporter plasmids (LXRα-WT and LXRα-Mut) and miR-155-5p mimic or its negative control for 48 h. The luciferase activity was then determined. **D**, **E** After 48 h of treatment with ox-LDL at 50 µg/mL, THP-1 macrophages were transfected with miR-155-5p mimic/inhibitor or their negative controls for 48 h. **D** Analysis of miR-155-5p expression by qRT-PCR. **E** Detection of LXRα expression by both qRT-PCR and western blot. Data are represented as the mean ± SD from three independent experiments. ﻿﻿****P < 0.0001; ns not significant.

Bioinformatics analyses (Targetscan and miRDB) for miRNA recognition sequences on LXRα revealed the presence of a putative miR-155-5p binding site (Fig. 4A). Further, the RNAhybrid database showed a lower free energy score (−19.0 kcal/mol), suggesting that miR-155-5p can bind stably to LXRα 3′ UTR (Fig. 4B). To further confirm the target prediction algorithms, we constructed a luciferase reporter plasmid containing either wild-type miR-155-5p binding site (LXRα-WT) or corresponding mutant (LXRα-Mut) (Fig. 4A). These plasmids were transfected into 293T cells together with miR-155-5p mimic or mimic control, followed by luciferase reporter assay. Co-transfection of LXRα-WT and miR-155-5p mimic markedly diminished the luciferase activity, while this effect disappeared when the miR-155-5p binding site was mutated (Fig. 4C). We then transfected THP-1 macrophage-derived foam cells with mimic control, miR-155-5p mimic, inhibitor control, and miR-155-5p inhibitor, respectively. In comparison with the control group, miR-155-5p mimic treatment elevated miR-155-5p levels by 7.1-fold, and miR-155-5p inhibitor suppressed miR-155-5p expression by 68% (Fig. 4D), showing a high transfection efficacy. Meanwhile, transfection with miR-155-5p mimic decreased the mRNA and protein levels of LXRα, while miR-155-5p inhibitor had an opposite effect (Fig. 4E). Overall, these data support the concept that miR-155-5p can directly interact with LXRα 3′ UTR and negatively modulate its expression at the post-transcriptional level.

### MiR-155-5p is required for CTRP12-induced upregulation of LXRα, ABCA1, and ABCG1 expression

**Fig. 5:**
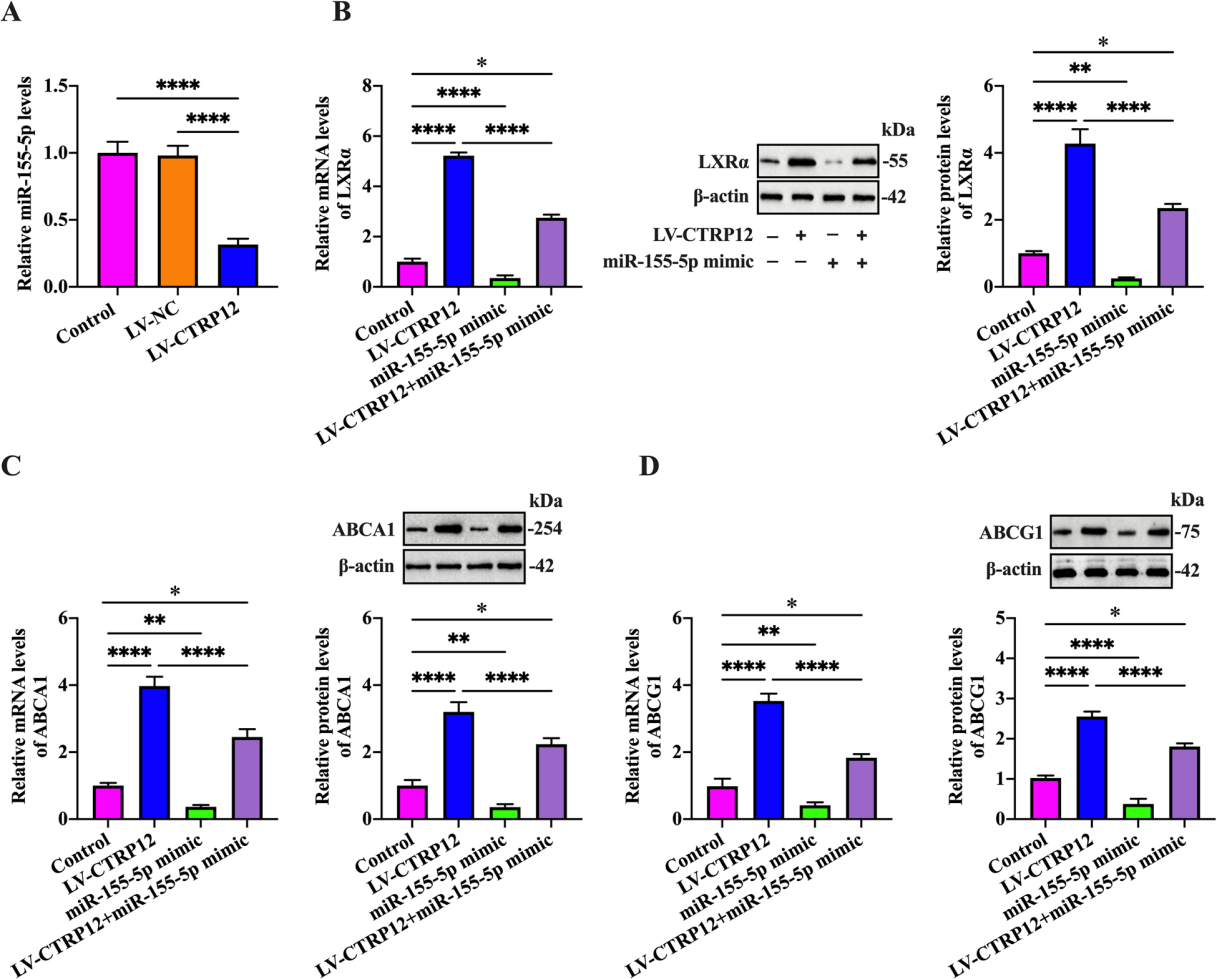
CTRP12-induced upregulation of LXRα, ABCA1, and ABCG1 is mediated by miR-155-5p. **A** THP-1 macrophages were treated with 50 µg/mL ox-LDL for 48 h and then transfected with PBS, LV-NC, or LV-CTRP12 for 72 h, followed by detection of miR-155-5p expression using qRT-PCR. **B**–**D** THP-1 macrophages were loaded with 50 µg/mL ox-LDL for 48 h, transfected with miR-155-5p mimic for another 48 h and then transduced with LV-CTRP12 for 72 h. The mRNA and protein levels of LXRα, ABCA1, and ABCG1 were detected by qRT-PCR and western blot, respectively. Data are represented as the mean ± SD from three independent experiments. ﻿﻿*P < 0.05, **P < 0.01, ****P < 0.0001.

The above studies have identified LXRα as a direct target of miR-155-5p. We speculated that CTRP12-induced upregulation of LXRα, ABCA1, and ABCG1 expression is likely mediated by miR-155-5p. To test this possibility, we first detected miR-155-5p expression using qRT-PCR in THP-1 macrophage-derived foam cells treated with LV-CTRP12. Our results indicated that CTRP12 overexpression markedly attenuated miR-155-5p levels (Fig. 5A). Subsequently, THP-1 macrophage-derived foam cells were transfected with miR-155-5p mimic prior to treatment with LV-CTRP12. As shown in Fig. 5B–D, treatment with LV-CTRP12 alone upregulated LXRα, ABCA1, and ABCG1 expression, and these effects were reversed by miR-155-5p mimic. These data suggest the involvement of miR-155-5p in CTRP12-induced enhancement of LXRα, ABCA1, and ABCG1 expression.

### CTRP12 attenuates inflammatory response through the miR-155-5p/LXRα pathway in macrophages

**Fig. 6:**
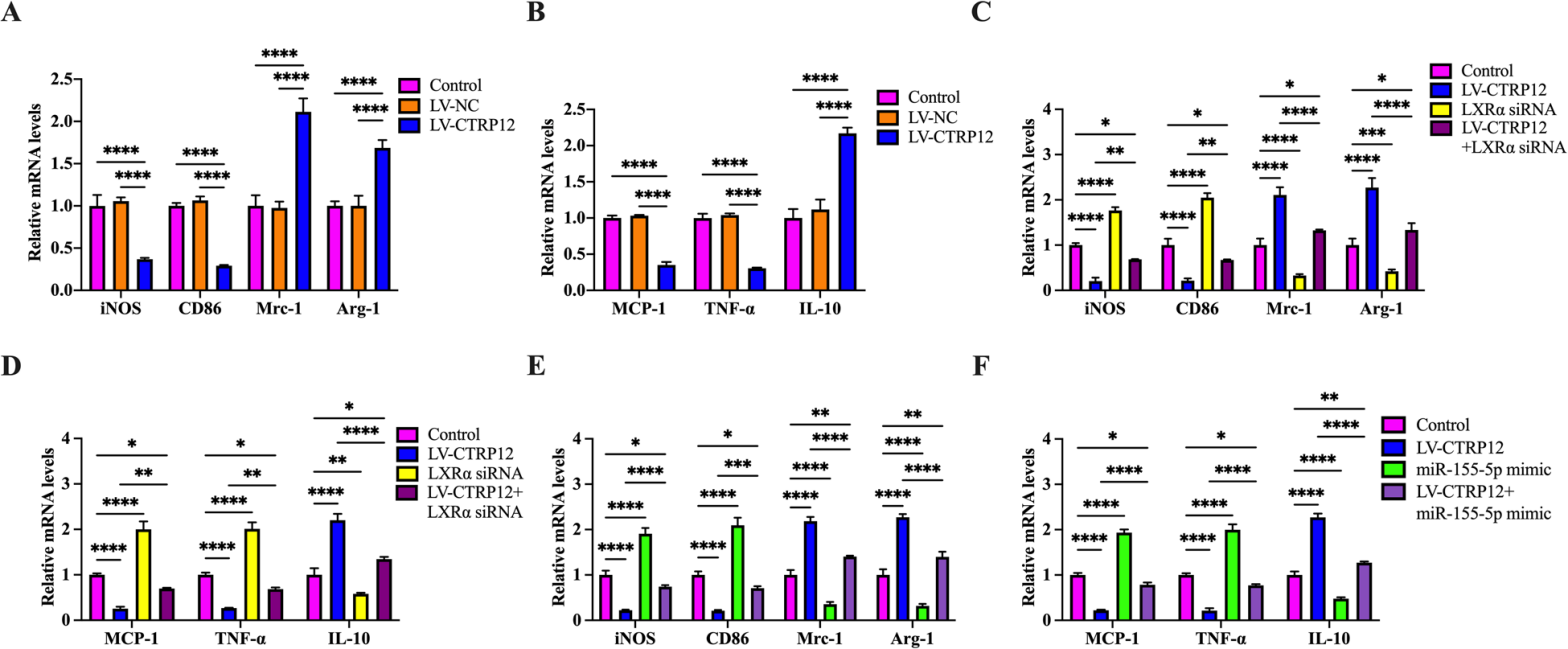
Effects of CTRP12 on macrophage polarization and inflammatory cytokine expression. **A**, **B** THP-1 macrophages were incubated with 50 µg/mL ox-LDL for 48 h, which was followed by transfection with PBS, LV-NC, or LV-CTRP12 for 72 h. The mRNA expression of iNOS, CD86, Mrc-1, Arg-1, MCP-1, TNF-α, and IL-10 was assayed using qRT-PCR. **C**–**F** THP-1 macrophages were loaded with 50 µg/mL ox-LDL for 48 h, transfected with LXRα siRNA or miR-155-5p mimic for another 48 h and then transduced with LV-CTRP12 for 72 h. The qRT-PCR was employed to measure the mRNA expression of iNOS, CD86, Mrc-1, Arg-1, MCP-1, TNF-α, and IL-10. Data are the mean ± SD from three independent experiments. ﻿﻿﻿﻿*P < 0.05, **P < 0.01, ***P < 0.001, ****P < 0.0001.

The atheroprotective role of LXRα is not only due to its influence on cholesterol efflux, but also on inflammatory response. Given CTRP12 as an activator of LXRα, we examined the impact of CTRP12 on macrophage polarization and inflammatory cytokine expression using qRT-PCR. As shown in Fig. 6A, treatment with LV-CTRP12 downregulated the mRNA expression of M1 markers inducible nitric oxide synthase (iNOS) and CD86 but upregulated the mRNA expression of M2 markers of mannose receptor C type 1 (Mrc-1) and arginase-1 (Arg-1) in THP-1 macrophage-derived foam cells. Consistently, CTRP12 overexpression decreased the mRNA levels of pro-inflammatory cytokines MCP-1 and TNF-α but increased the mRNA levels of anti-inflammatory cytokine IL-10 (Fig. 6B). The effects of CTRP12 on the mRNA expression of iNOS, CD86, Mrc-1, Arg-1, MCP-1, TNF-α, and IL-10 was significantly reversed by LXRα siRNA (Fig. 6C, D) and miR-155-5p mimic (Fig. 6E, F). To further confirm the data obtained using THP-1 macrophage-derived foam cells, we isolated MPMs from apoE^−/−^ mice and found that CTRP12 overexpression attenuated the mRNA levels of iNOS, CD86, MCP-1, and TNF-α but elevated the mRNA levels of Mrc-1, Arg-1, and IL-10 (Supplementary Fig. 2A, B). Pretreatment with LXRα siRNA or miR-155-5p mimic also diminished the influence of CTRP12 on the mRNA expression of iNOS, CD86, Mrc-1, Arg-1, MCP-1, TNF-α, and IL-10 (Supplementary Fig. 2C-F). Collectively, these observations suggest that CTRP12 exerts an anti-inflammatory effect by promoting M2 macrophage polarization via the miR-155-5p/LXRα signaling pathway.

### CTRP12 inhibits the development of atherosclerosis in apoE^−/−^ mice

**Fig. 7:**
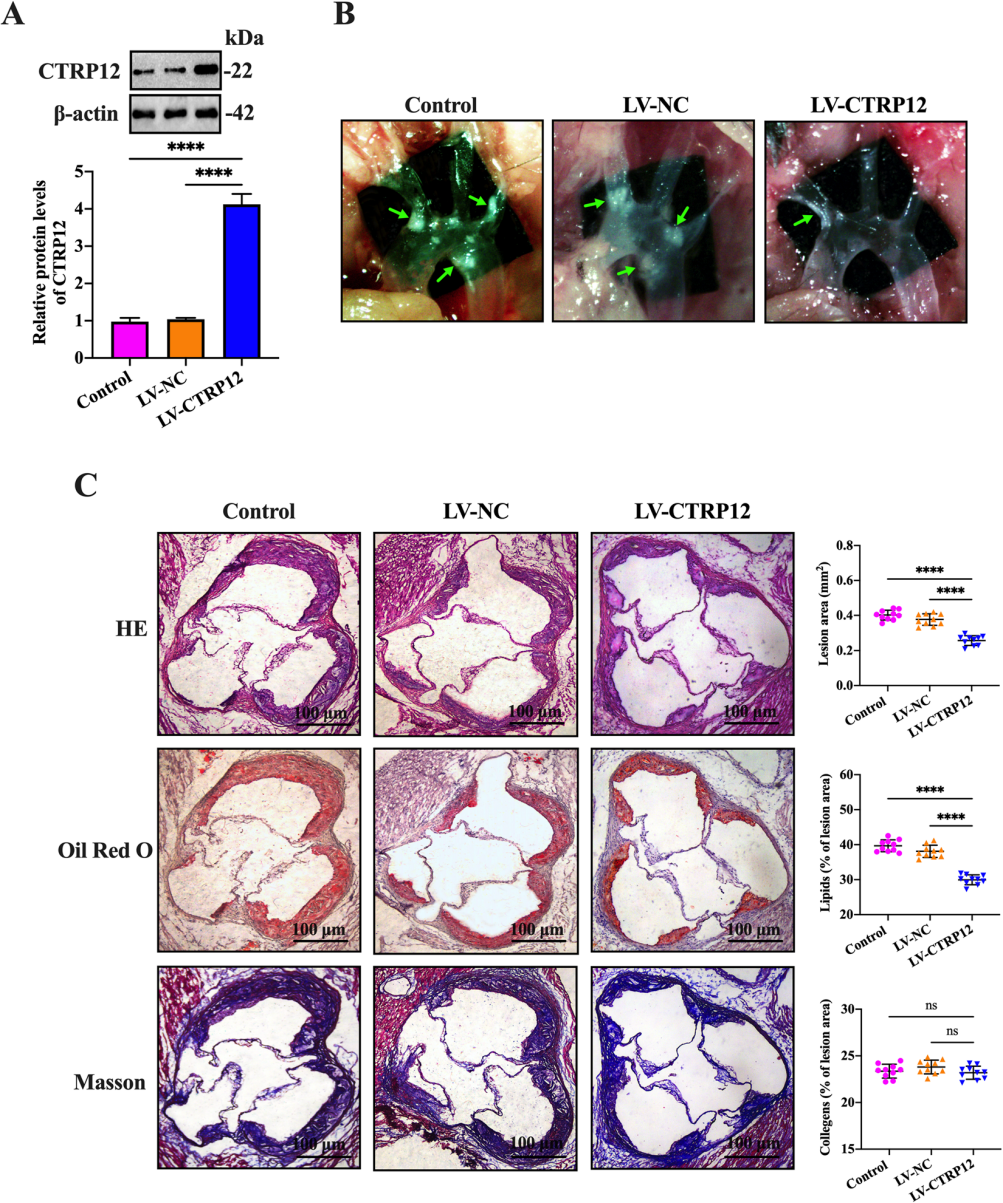
CTRP12 inhibits atherosclerosis in apoE^−/−^ mice. **A**–**C** Western diet-fed apoE^−/−^ mice were injected via the tail vein with PBS, LV-NC, or LV-CTRP12 (*n* = 15 in each group). **A** Analysis of CTRP12 expression in the aortas by western blot (*n* = 10). ﻿**B** The plaques (green arrows) in the aortic arch under a stereoscopic microscope (*n* = 10); **C** Cryosections of the aortic root were stained with HE, Oil Red O, or Masson﻿, followed by quantification of lesion area, lipid accumulation and collagen contents using Image-Pro Plus 7.0 software﻿ (*n* = 10). Scale bar = 100 μm. ﻿Data are represented as the mean ± SD. ****P < 0.0001; ns not significant.

Finally, to determine the role of CTRP12 in atherosclerosis in vivo, apoE^−/−^ mice were fed a ﻿Western diet for 12 weeks and simultaneously injected with PBS, LV-NC or LV- CTRP12. At the end of the study, about 4.1-fold elevation of CTRP12 protein levels was observed in the aortas from apoE^−/−^ mice transduced with LV-CTRP12 compared with control mice (Fig. 7A). Meanwhile, the number and size of atherosclerotic lesions in the aortic arch regions was significantly decreased in LV-CTRP12-treated group compared with control group (Fig. 7B). HE, Oil Red O and Masson staining of cross-sections of the aortic root showed that CTRP12 overexpression markedly decreased lesion area and inhibited lipid deposition, with no impact on collagen content (Fig. 7C). These in vivo results indicate that CTRP12 plays a protective role in the development of atherosclerosis.

**Fig. 8:**
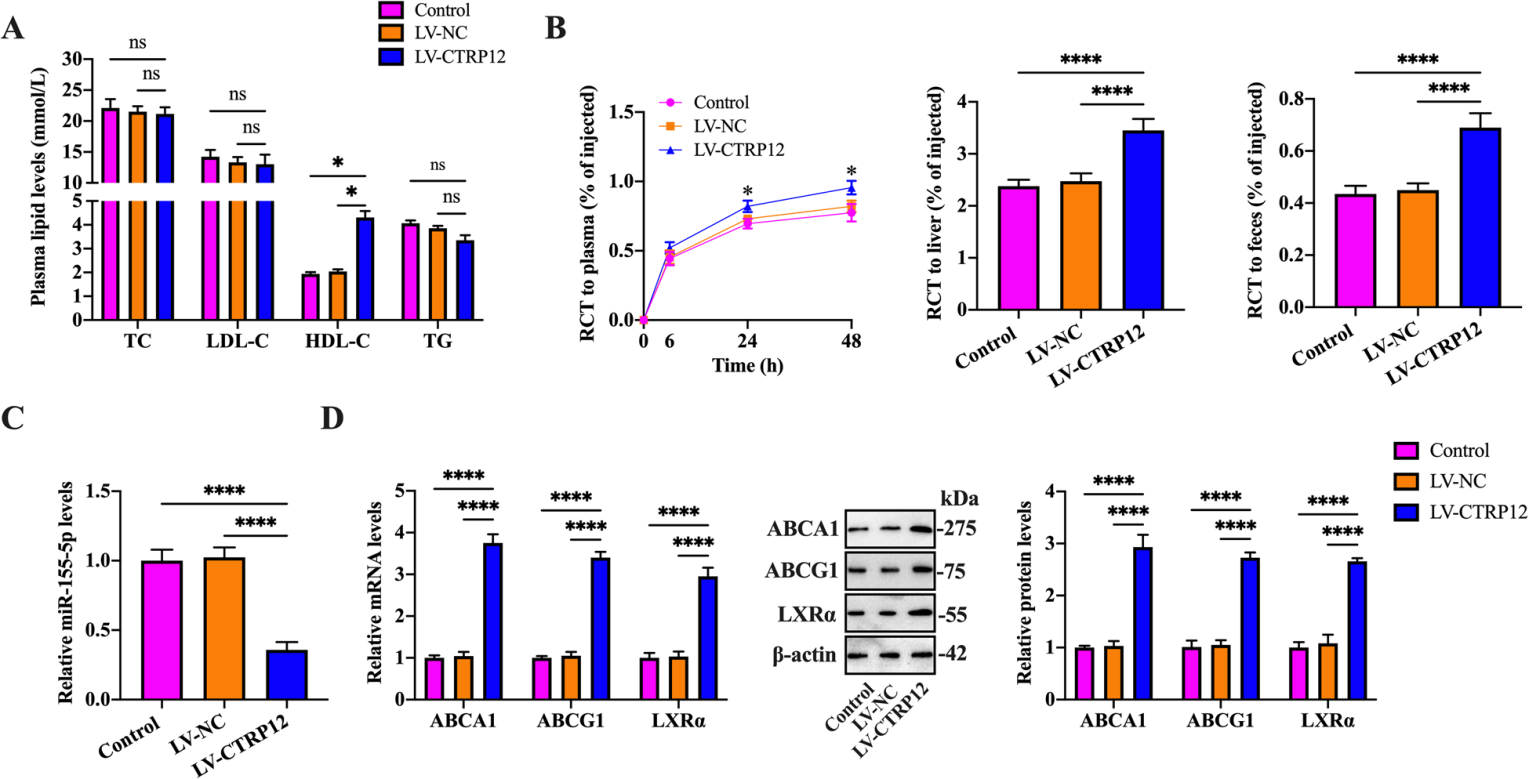
Effects of CTRP12 on plasma HDL-C levels and RCT in apoE^−/−^ mice. **A** ﻿Plasma levels of TC, TG, HDL-C, and LDL-C were determined using the commercial kits ﻿(*n* = 10). **B** Mice were injected intraperitoneally with [^3^H]-cholesterol-labeled J774 macrophages. ﻿The radioactivity in the plasma, liver, and feces were assessed by a liquid scintillation counter (*n* = 5). **C**, **D** Detection of miR-155-5p, LXRα, ABCA1, and ABCG1 expression in the aortas by qRT-PCR and western blot (*n* = 10). Data are represented as the mean ± SD. ﻿*P < 0.05, ****P < 0.0001; ns not significant.

To elucidate the underlying mechanisms by which CTRP12 protects against atherosclerosis in vivo, we first detected the cholesterol and TG concentrations in MPMs. Our results revealed that ox-LDL loading enhanced the amounts of TC, FC, CE, and TG, which was prevented by LV-CTRP12 transduction (Supplementary Fig. 3A, B). Subsequently, we examined the effects of CTRP12 on plasma lipid profile and RCT. Figure 8A shows that injection of LV-CTRP12 elevated HDL-C levels but had no impact on TC, LDL-C, and TG levels. Consistently, apoE^−/−^ mice injected with LV-CTRP12 exhibited a significant increase of [^3^H]-cholesterol content in the plasma, liver, and feces (Fig. 8B), suggesting a promotive effect of CTRP12 on RCT. In line with the findings of in vitro experiments, LV-CTRP12 injection decreased miR-155-5p levels but enhanced the expression of LXRα, ABCA1, and ABCG1 in the aortas of apoE^−/−^ mice (Fig. 8C, D). Meanwhile, CTRP12 overexpression downregulated iNOS, CD86, MCP-1, and TNF-α expression but upregulated Mrc-1, Arg-1, and IL-10 expression (Supplementary Fig. 4A, B). CTRP12 overexpression also led to a significant decrease in serum MCP-1 and TNF-α levels and an increase in serum IL-10 levels (Supplementary Fig. 4C). Taken together, all these data demonstrate that the antiatherogenic action of CTRP12 is attributed to its abilities to promote RCT and suppress vascular inflammation.

## Discussion

As a paralog of adiponectin, CTRP12 is composed of a signal peptide, a short N-terminal domain, a collagen-like domain and a globular C1q-like domain. Adiponectin is known to protect against atherosclerosis in animal models^32,33^. Although CTRP12 was reported to participate in the occurrence and development of cardiovascular disease^22–24^, its role in atherogenesis is still largely unknown. Our in vivo experiments demonstrated that aortic plaque burden was significantly lower in apoE^−/−^ mice overexpressing CTRP12 than that in apoE^−/−^ littermates. This is the first study to report an association between CTRP12 and atherosclerosis.

Lipid metabolism disorder plays a critical role in the pathogenesis of atherosclerosis. An epidemiological survey showed that a 1 mg/dL elevation of plasma HDL-C levels decreases the risk of coronary heart disease in men by 2% and women by 3%^34^. RCT, a process in which excessive peripheral cholesterol is transported by HDL to the liver for excretion into the bile and feces, is proposed to be a major mechanism for the antiatherogenic action of HDL. In our previous studies, administration of mangiferin, a xanthonoid extracted from *Salacia oblonga*, is protective against atherosclerosis by elevating plasma HDL-C levels and promoting RCT in apoE^−/−^ mice^35^. CTRP12 increases insulin sensitivity and improves glucose metabolism^36,37^. Partial deficiency of CTRP12 in male mice fed a high-fat diet impairs lipid clearance from the body and consequently augments hepatic cholesterol content^38^. In patients with coronary artery disease, circulating CTRP12 concentration is positively correlated with HDL-C levels^24^. Here, we found that overexpression of CTRP12 led to a significant increase in circulating HDL-C levels and RCT efficiency, which was accompanied by decreased lipid deposition within the plaques. Thus, CTRP12 exerts a beneficial impact on lipid metabolism, which contributes to its atheroprotective effect.

Excessive lipid deposition in macrophages results in foam cell formation, which is regarded as a critical event during atherogenesis. There is increasing evidence that adiponectin inhibits lipid accumulation and subsequent transformation of macrophages into foam cells^39–41^. Similarly, treatment with recombinant CTRP12 suppresses lipogenesis in rat H4IIE hepatoma cells^42^. In this study, we found that overexpression of CTRP12 significantly reduced intracellular lipid droplets and decreased cholesterol and TG contents in macrophages. These data suggest that CTRP12 contributes to alleviation of macrophage lipid accumulation.

ABCA1 and ABCG1 belong to the members of the ABC superfamily and promote cholesterol efflux to apoA-I and HDL, respectively. It is estimated that they are responsible for ~70% of cholesterol release from lipid-loaded macrophages^43^. Our groups and others have revealed that administration of fargesin, mangiferin, or polydatin inhibits macrophage lipid accumulation and atherosclerotic plaque formation by facilitating ABCA1- and ABCG1-dependent cholesterol efflux^6,35,44^. SR-A and CD36 are the members of the scavenger receptor family and account for 75–90% of ox-LDL internalization by macrophages^45^. Extensive studies have demonstrated that prevention of SR-A and/or CD36 expression contributes to alleviation of lipid deposition in macrophages^46–48^. In this study, we observed that treatment with LV-CTRP12 markedly upregulated ABCA1 and ABCG1 expression in THP-1 macrophage-derived foam cells, which was accompanied by a significant increase in cholesterol efflux toward apoA-I and HDL. Thus, promotion of ABCA1- and ABCG1-mediated cholesterol efflux is a key mechanism by which CTRP12 suppresses macrophage lipid accumulation and mitigates atherosclerosis. Notably, Dil-ox-LDL internalization and the expression of SR-A and CD36 was not affected by CTRP12 overexpression, suggesting that CTRP12-induced alleviation of lipid accumulation is not due to decreased cholesterol uptake.

LXRα is a nuclear hormone receptor that is activated by oxysterols and endogenous oxidative metabolites of cholesterol. After forming an obligate heterodimer with retinoid X receptor, LXRα binds to LXR-response elements in the promoter of *ABCA1* and *ABCG1* genes to stimulate their transcription^49^. Studies from our group and others have revealed that angiopoietin-1, pregnancy-associated plasma protein-A, and homocysteine inhibit ABCA1- and ABCG1-dependent cholesterol efflux from lipid-loaded macrophages and aggravate atherosclerosis in apoE^−/−^ mice by downregulating LXRα expression^50–52^. As a pleiotropic miRNA, miR-155-5p is linked to metabolic diseases, such as diet-induced obesity^53^ and diabetes^54^. A recent study showed that metastasis-associated lung adenocarcinoma transcript 1, a long noncoding RNA (lncRNA), enhances nuclear factor I/A expression by competitively binding to miR-155-5p, thereby blocking ox-LDL-stimulated dendritic cell maturation and attenuating atherosclerosis in apoE^−/−^ mice^55^. This finding reveals a proatherogenic effect of miR-155-5p. In the current studies, we identified LXRα as a direct target of miR-155-5p, as evidenced by bioinformatics prediction and luciferase reporter assay. Transduction with LV-CTRP12 was shown to upregulate LXRα expression and attenuate miR-155-5p levels in THP-1 macrophage-derived foam cells and the aortas from apoE^−/−^ mice. Further, pretreatment with LXRα siRNA or miR-155-5p mimic reduced the effects of LV-CTRP12 on ABCA1 and ABCG1 expression, suggesting that CTRP12-induced enhancement of ABCA1 and ABCG1 expression and promotion of cholesterol efflux is mediated by the miR-155-5p/LXRα pathway.

Atherosclerosis is regarded as a lipid-driven inflammatory disease. Under inflammatory conditions, macrophages can polarize into two major phenotypes called classically activated M1 macrophages and alternatively activated M2 macrophages. M1 macrophages secrete pro-inflammatory cytokines and promote the development of atherosclerosis, while M2 macrophages contribute to inflammation resolution and play an atheroprotective role^56,57^. In LPS-stimulated cardiomyocytes, overexpression of CTRP12 blocks the generation and release of pro-inflammatory cytokines^58^. Treatment with recombinant CTRP12 decreases the expression of MCP-1, TNF-α and IL-6 in LPS-treated macrophages^59^. Injection of miR-155-5p agomir increases TNF-α, IL-1β, and IL-6 amounts in the ventral midbrain of Parkinson’s disease mice^60^. Additionally, LXRα overexpression induces M1 to M2 phenotypic transition in macrophages^61^. Our results showed that CTRP12 polarized macrophages towards an M2 phenotype and attenuated vascular inflammation through the miR-155-5p/LXRα pathway, thereby providing another important mechanism for its antiatherogenic action. A growing body of evidence has indicated that lncRNAs are pivotal regulators of atherogenesis^62,63^. More recently, Wang et al. reported that the expression levels of CTBP1-AS2, a lncRNA, were significantly decreased in the serum of patients with atherosclerosis, and its overexpression inhibits proliferation and promotes autophagy in human aortic smooth muscle cells challenged with ox-LDL^64^. Another study revealed that CTBP1-AS2 sponges miR-155-5p to suppress pro-inflammatory cytokine production induced by high glucose in human glomerular mesangial cells^65^. Thus, it is possible that CTBP1-AS2 is involved in CTRP12-induced downregulation of miR-155-5p expression. Future studies will be needed to confirm this possibility.

In summary, the present study has demonstrated a novel role of macrophage CTRP12 in inhibiting the development of atherosclerosis and uncovered a novel mechanism underlying the regulation of ABCA1 and ABCG1. CTRP12 decreases miR-155-5p levels and then increases LXRα expression, which promotes ABCA1- and ABCG1-dependent cholesterol efflux and alleviates inflammatory response. These data extend our understanding for the biological functions of CTRP12 and provide a potential therapeutic target for atherosclerotic cardiovascular disease.

## Supplementary information

Supplementary Figure Legends

Supplementary Figure 1

Supplementary Figure 2

Supplementary Figure 3

Supplementary Figure 4

Supplementary table 1
