# Supplementary material for: CTRP12 ameliorates atherosclerosis by promoting cholesterol efflux and inhibiting inflammatory response via the miR-155-5p/LXRα pathway
Source: Cell Death Dis. 2021 Mar 10;12(3):254. doi: 10.1038/s41419-021-03544-8 (PMC7947013; doi:10.1038/s41419-021-03544-8)
Supplement: Supplementary file 1 — Supplementary Figure Legends [file 41419_2021_3544_MOESM1_ESM.docx]

**Supplementary Fig. 1 CTRP12 promotes cholesterol efflux from THP-1 macrophages loaded with ac-LDL.** THP-1 macrophages were loaded with 50 µg/mL ac-LDL and 5 µCi/mL [^3^H]-cholesterol for 24 h. Then, cells were cultured in fresh medium containing 10 µM avasimibe with or without 0.5 mM 8-Br-cAMP. The efficiency of cholesterol efflux to BSA, apoA-I and HDL was detected using a liquid scintillation counter. Data are the mean ± SD from three independent experiments. ﻿*****P* < 0.0001; ns, not significant.

**Supplementary Fig. 2 Effects of CTRP12 on macrophage polarization and inflammatory cytokine expression in MPMs. A, B** MPMs were isolated from apoE^-/-^ mice injected with PBS, LV-NC or LV- CTRP12 (n=5 in each group) and then loaded with 50 µg/mL ox-LDL for 48 h. The expression of iNOS, CD86, Mrc-1, Arg-1, MCP-1, TNF-α and IL-10 was checked by qRT-PCR. **C-F** MPMs were transfected with LXRα siRNA or miR-155-5p mimic for 48 h after ox-LDL loading, followed by detection of iNOS, CD86, Mrc-1, Arg-1, MCP-1, TNF-α and IL-10 expression using qRT-PCR. Data are represented the mean ± SD. **P* < 0.05, ***P* < 0.01, ****P* < 0.001, *****P* < 0.0001.

**Supplementary Fig. 3 CTRP12 decreases the cholesterol and TG contents in MPMs. A, B** MPMs were isolated from apoE^-/-^ mice injected with PBS, LV-NC or LV-CTRP12 (n=5 ), followed by incubation with or without 50 µg/mL ox-LDL for 48 h. **A** HPLC was used to determine intracellular TC, FC and CE levels. **B.** Detection of intracellular TG concentration using a commercial kit. Data are expressed as the mean ± SD. **P* < 0.05, ***P* < 0.01, *****P* < 0.0001.

**Supplementary Fig. 4 CTRP12 attenuates inflammatory response *in vivo*. A, B** The mRNA levels of iNOs, CD86, Mrc-1, Arg-1, MCP-1, TNF-α and IL-10 in the aortas of apoE^-/-^ mice were measured by qRT-PCR (n=10). **C** ELISA was employed to measure serum MCP-1, TNF-α and IL-10 levels (n=10). Data are expressed as the mean ± SD. **P* < 0.05, ***P* < 0.01, ****P* < 0.001, *****P* < 0.0001.

**Supplementary Table 1.** The primer sequences used in qRT-PCR.
