# Supplementary figures and images for: CTRP12 ameliorates atherosclerosis by promoting cholesterol efflux and inhibiting inflammatory response via the miR-155-5p/LXRα pathway
Source: Cell Death Dis. 2021 Mar 10;12(3):254. doi: 10.1038/s41419-021-03544-8 (PMC7947013; doi:10.1038/s41419-021-03544-8)

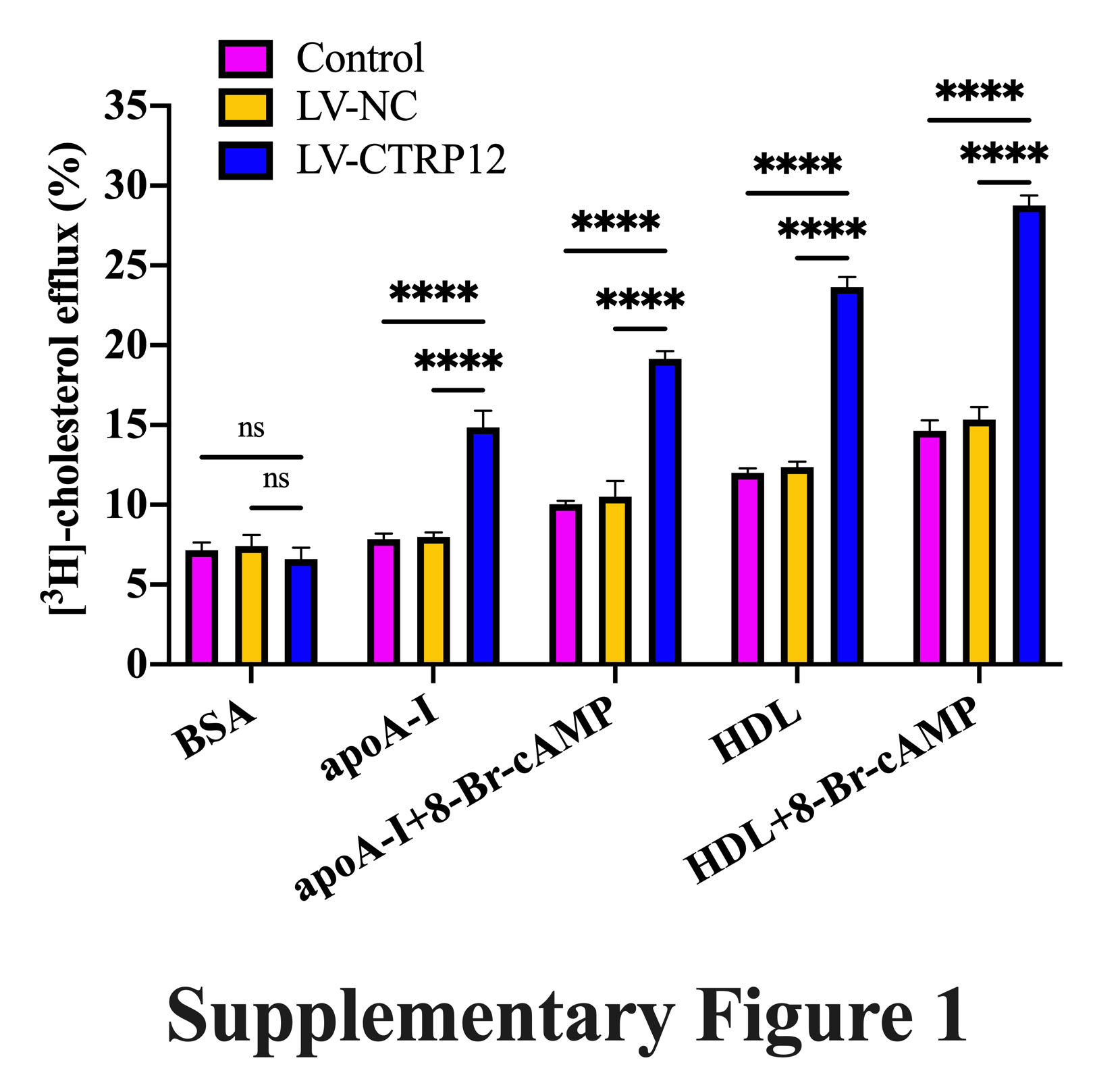

Supplement: Supplementary file 2 — Supplementary Figure 1 [file 41419_2021_3544_MOESM2_ESM.tif]

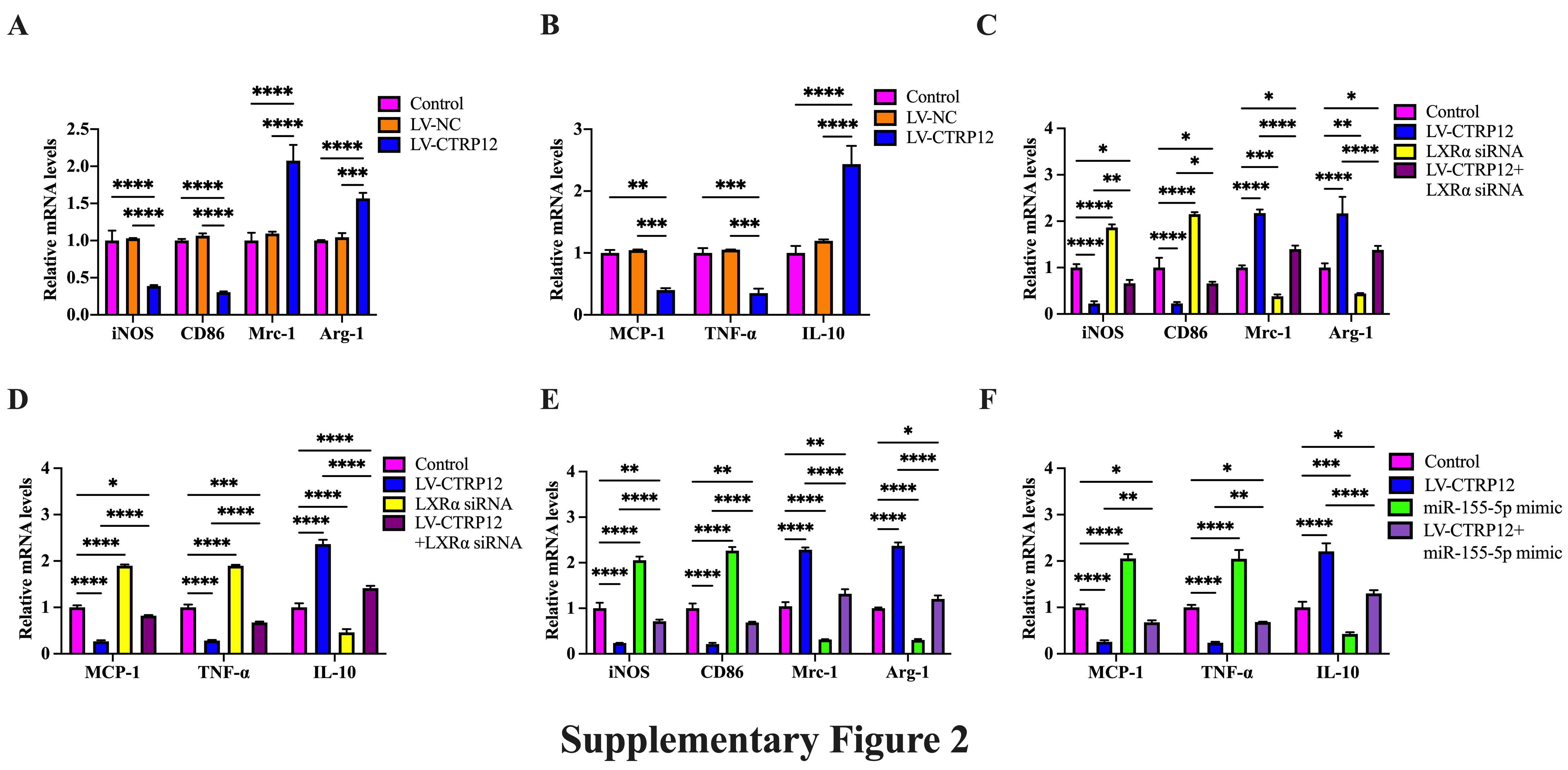

Supplement: Supplementary file 3 — Supplementary Figure 2 [file 41419_2021_3544_MOESM3_ESM.tif]

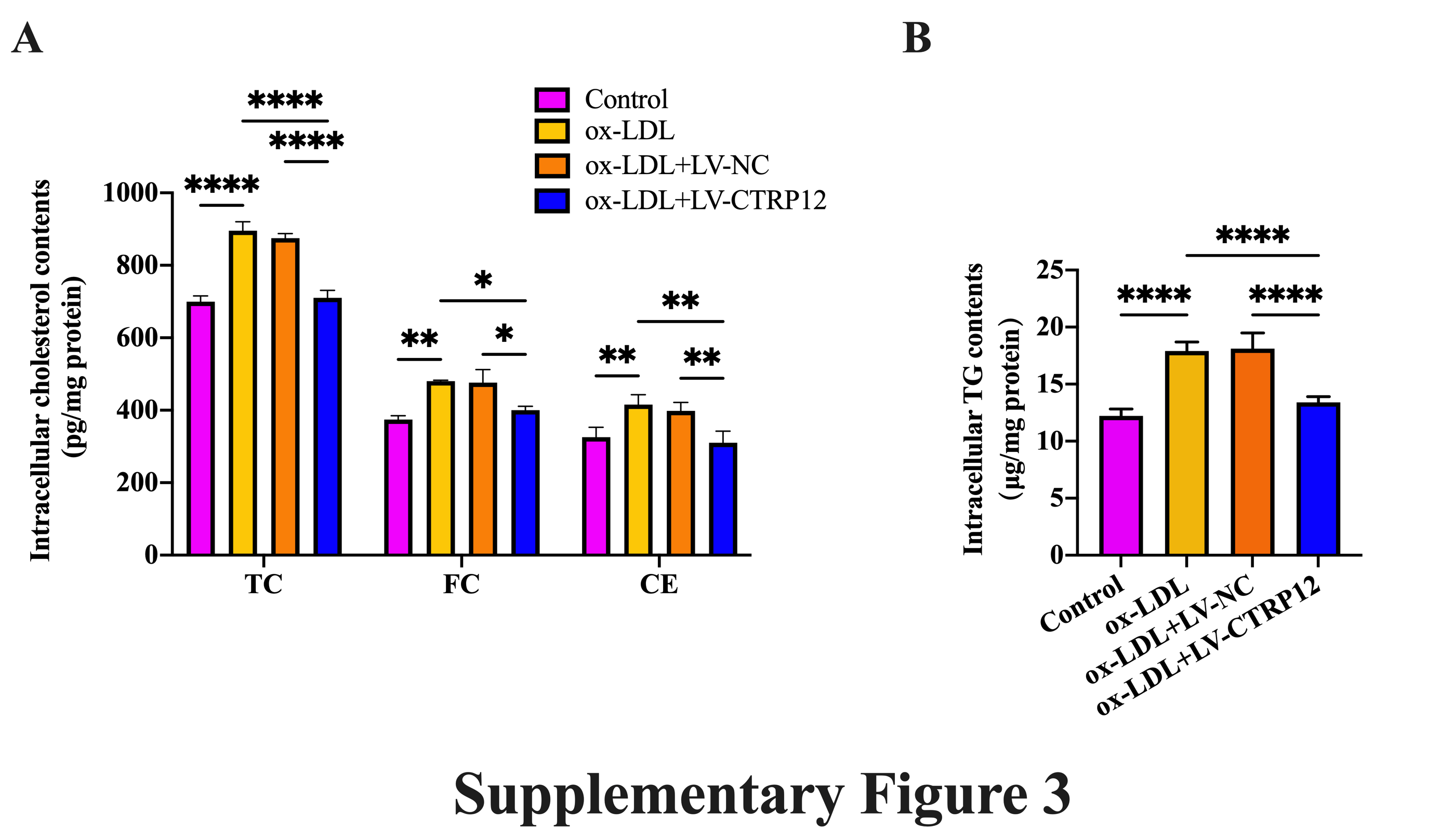

Supplement: Supplementary file 4 — Supplementary Figure 3 [file 41419_2021_3544_MOESM4_ESM.tif]

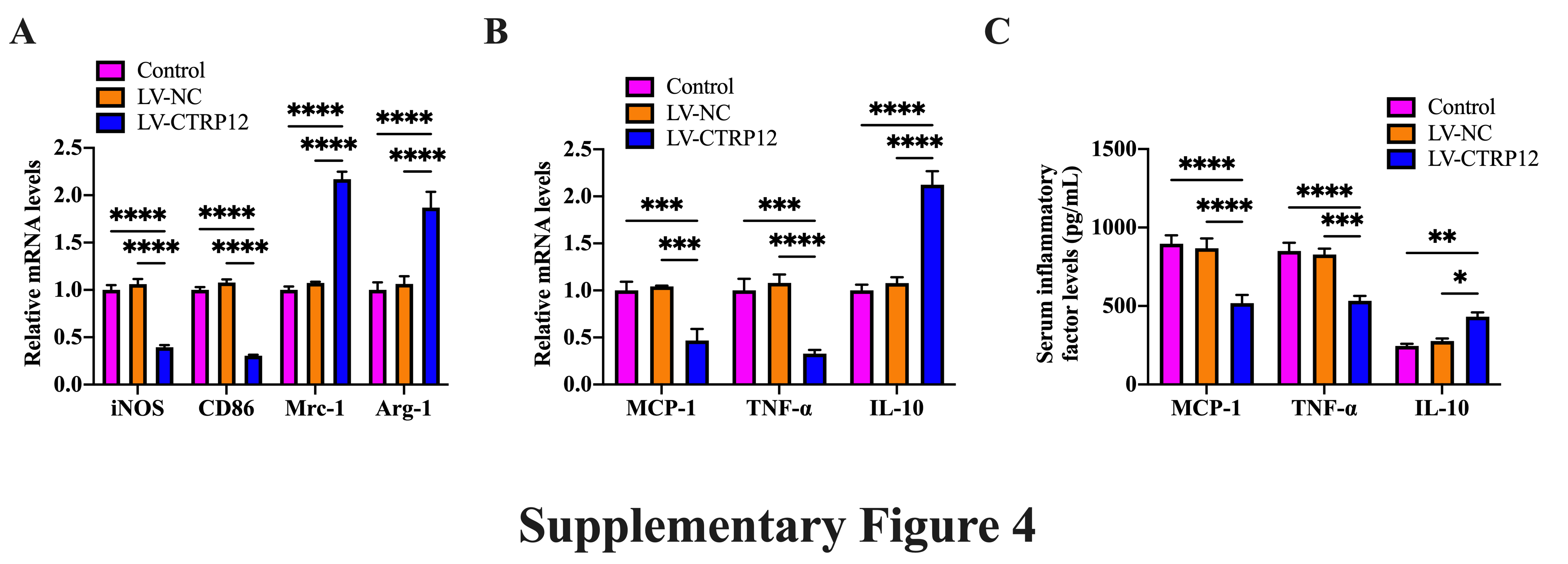

Supplement: Supplementary file 5 — Supplementary Figure 4 [file 41419_2021_3544_MOESM5_ESM.tif]
