# Supplementary material for: CTRP12 ameliorates atherosclerosis by promoting cholesterol efflux and inhibiting inflammatory response via the miR-155-5p/LXRα pathway
Source: Cell Death Dis. 2021 Mar 10;12(3):254. doi: 10.1038/s41419-021-03544-8 (PMC7947013; doi:10.1038/s41419-021-03544-8)
Supplement: Supplementary file 6 — Supplementary table 1 [file 41419_2021_3544_MOESM6_ESM.docx]

**Supplementary Table 1.**

| **Gene** | **Sense** | **Anti-sense** |
| --- | --- | --- |
| CTRP12 (Human) | 5*'*-AGCTGCATGGTTTCCAGGCTCC-3*'* | 5*'*-CTGGCAGAGAACTGGAAGATGC-3*'* |
| CTRP12 (Mouse) | 5*'*-AGCTGTTTGCGCGTGTGGATTC-3*'* | 5*'*-CGTCGGACAAAGTTCAACCACG-3*'* |
| ABCA1 (Human) | 5*'*-GTCCTCTTTCCCGATTATCTGG-3*'* | 5*'*-CACTCACTCTCGCTCGCAAT-3*'* |
| ABCA1 (Mouse) | 5*'*-GGGTGGTGTTCTTCCTCATTAC-3*'* | 5*'*-GAATGACGAGGATGAGGATGTG-3*'* |
| ABCG1 (Human) | 5*'*-TGTTCATCAGCGTGCACTTC-3*'* | 5*'*-AGGGCTCAAGCATTGTCATC-3*'* |
| ABCG1 (Mouse) | 5*'*-AGGTCTCAGCCTTCTAAAGTTCCTC-3*'* | 5*'*-TCTCTCGAAGTGAATGAAATTTATCG-3*'* |
| SR-A (Human) | 5*'*-TTTGATGCTCGCTCAATGACA-3*'* | 5*'*-GCTGCCACTATTCCAATGAGAG-3*'* |
| SR-A (Mouse) | 5*'*-TGGTCCACCTGGTGCTCC-3*'* | 5*'*-ACCTCCAGGGAAGCCAATTT-3*'* |
| CD-36 (Human) | 5*'*-TGCTCATCTATACACGGTTACC-3*'* | 5*'*-TGCTCATCTATACACGGTTACC-3*'* |
| CD-36 (Mouse) | 5*'*-CCCAGATGCACCATGGGCTTGGCAA-3*'* | 5*'*-AAGCTCGTGCGGCCCAGGTACT-3*'* |
| LXRα (Human) | 5*'*-CGATCGAGGTGATGCTTCTG-3*'* | 5*'*-GGCAAAGTCTTCCCGGTTAT-3*'* |
| LXRα (Mouse) | 5*'*-AGGAGTGTCGACTTCGCAAA-3*'* | 5*'*-CTCTTCTTGCCGCTTCAGTTT-3*'* |
| miR-155-5p (Human) | 5*'*-GAGGGTTAATGCTAATCGTGATAGG-3*'* | 5*'*-GCACAGAATCAACACGACTCACTAT-3*'* |
| miR-155-5p (Mouse) | 5*'*-TTAATGCTAATTGTGATAGGGGT-3*'* | 5*'*-GCAGGGTCCGAGGTATTC-3*'* |
| TNF-α (Human) | 5*'*- CTCTTCTGCCTGCTGCACTTTG-3*'* | 5*'*- ATGGGCTACAGGCTTGTCACTC-3*'* |
| TNF-α (Mouse) | 5’-ACCCTCACACTCAGATCATCTT-3’ | 5’-GGTTGTCTTTGAGATCCATGC-3’ |
| MCP-1 (Human) | 5’-AGAATCACCAGCAGCAAGTGTCC-3’ | 5’-TCCTGAACCCACTTCTGCTTGG-3’ |
| MCP-1 (Mouse) | 5’-GCAGCAGGTGTCCCAAAGAA-3’ | 5’-ATTTACGGGTCAACTTCACATTCAA-3’ |
| IL-10 (Human) | 5’-TCTCCGAGATGCCTTCAGCAGA-3’ | 5’-TCAGACAAGGCTTGGCAACCCA-3’ |
| IL-10 (Mouse) | 5’-CGGGAAGACAATAACTGCACCC-3’ | 5’-CGGTTAGCAGTATGTTGTCCAGC-3’ |
| iNOS (Human) | 5′-CCGAGCAAGTCCGAAACAAG-3′ | 5′-CCGAGCAAGTCCGAAACAAG-3′ |
| iNOS (Mouse) | 5′-CAGAGGACCCAGAGACAAGC-3′ | 5′- TGCTGAAACATTTCCTGTGC-3′ |
| CD86 (Human) | 5′-TGCTCATCTATACACGGTTACC-3′ | 5′- TGCTCATCTATACACGGTTACC-3′ |
| CD86 (Mouse) | 5′-CCCAGATGCACCATGGGCTTGGCAA-3′ | 5′- AAGCTCGTGCGGCCCAGGTACT-3′ |
| Mrc-1 (Human) | 5′-TACCCCTGCTCCTGGTTTTT-3′ | 5′-CAGCGCTTGTGATCTTCATT-3′ |
| Mrc-1 (Mouse) | 5′-GGCGAGCATCAAGAGTAAAGA-3′ | 5′-TTTGGTTGGGACTGACCTATG -3′ |
| Arg-1 (Human) | 5′-TGGAAGTGAACCCATCCCTG-3′ | 5′-CCGAGCAAGTCCGAAACAAG-3′ |
| Arg-1 (Mouse) | 5′-GCGCCTTTCTCAAAAGGACAGC-3′ | 5′-TGGCTTTCCCCACAGACCGTG-3′ |
| β-actin (Human) | 5*'*-GACCTCTATGCCAACACAGT-3*'* | 5*'*-AGTACTTGCGCTCAGGAGGA-3*'* |
| β-actin (Mouse) | 5*'*-TGGCACCCAGCACAATGAA-3*'* | 5*'*-CTAAGTCATAGTCCGCCTAGAAGCA-3*'* |
| U6 (Human) | 5*'*-GAGGGTTAATGCTAATCGTGATAGG-3*'* | 5*'*-GCACAGAATCAACACGACTCACTAT-3*'* |
| U6 (Mouse) | 5*'*-CGCAAGGATGACACGCAAAT-3*'* | 5*'*-GTGCAGGGTCCGAGGTATTC-3*'* |
